# Ligand and Structure-Based Virtual Screening in Combination, to Evaluate Small Organic Molecules as Inhibitors for the XIAP Anti-Apoptotic Protein: The Xanthohumol Hypothesis

**DOI:** 10.3390/molecules27154825

**Published:** 2022-07-28

**Authors:** Angeliki Mavra, Christos C. Petrou, Manos C. Vlasiou

**Affiliations:** Department of Life & Health Sciences, University of Nicosia, Nicosia 2417, Cyprus; mavra.a@live.unic.ac.cy (A.M.); petrou.c@unic.ac.cy (C.C.P.)

**Keywords:** molecular docking, molecular dynamics, pharmacophore, molecular modeling, XIAP protein, protein inhibitor, ADMET studies, anticancer activity

## Abstract

Herein, we propose two chalcone molecules, (E)-1-(4-methoxyphenyl)-3-(p-tolyl) prop-2-en-1-one and (E)-3-(4-hydroxyphenyl)-1-(2,4,6-trihydroxyphenyl) prop-2-en-1-one, based on the anticancer bioactive molecule Xanthohumol, which are suitable for further in vitro and in vivo studies. Their ability to create stable complexes with the antiapoptotic X-linked IAP (XIAP) protein makes them promising anticancer agents. The calculations were based on ligand-based and structure-based virtual screening combined with the pharmacophore build. Additionally, the structures passed Lipinski’s rule for drug use, and their reactivity was confirmed using density functional theory studies. ADMET studies were also performed to reveal the pharmacokinetic potential of the compounds. The candidates were chosen from 10,639,400 compounds, and the docking protocols were evaluated using molecular dynamics simulations.

## 1. Introduction

In recent decades, researchers have paid much attention to the antitumor effect of the prenylated chalcone called Xanthohumol (XN). More specifically, the anticancer activity of this molecule has been marked by intracellular ROS induction, endoplasmic reticulum stress induction, and disruption of the BIG3-PHB2 interaction [1]. The BIG3-PHB2 interaction happens between the A-inhibited guanine nucleotide-exchange protein 3 (BIG3) and prohibitin 2 (PHB2) in the cytoplasm of breast cancer cells [2,3]. Additionally, XN inhibits DNA synthesis, arresting the cancer cell cycle at the S phase [4]. The chemical structure of this flavonoid consists of trans-configured A, and B aromatic rings joined through a three-carbon, unsaturated carbonyl system substituted by hydroxyl groups, a methoxy group, and a prenyl unit [5]. 

Programmed cell death is one of the most common cancer therapies in tumor cells. Defects in the pro-apoptotic death regulators, such as BH3-only proteins, cause chemotherapy failure [6]. The inhibition of apoptosis proteins (IAP) through apoptosis regulators is characterized by the presence of three domains known as baculoviral IAP repeat (BIR) domains [7,8]. Among these IAP proteins, cellular IAP-1 (cIAP-1) and cIAP-2 play a critical role in the regulation of tumor necrosis factor (TNF) receptor-mediated apoptosis, while X-linked IAP (XIAP) is a central regulator of both deaths’ receptor-mediated and mitochondria-mediated apoptosis pathways [9]. XIAP inhibits apoptosis by suppressing caspase activity, whereas the third BIR domain (BIR3) of XIAP selectively targets an initiator caspase-9, the BIR2 domain, and the linker immediately preceding it, inhibiting effector caspase-3/caspase-7. XIAP and cIAP-1 are highly expressed in cancers of diverse tumor types and are considered attractive cancer therapeutic targets [10,11]. 

Structure-based pharmacophore (SBP) design and hybrid virtual screening protocols can be used to detect novel Xanthohumol-based lead compounds based on changes in the chemical scaffold [12]. Pharmacophore query is widely applied in database screening. To date, the ligand-based pharmacophore model has been utilized frequently. Nowadays, with large numbers of protein structures being elucidated, the application of the structure-based pharmacophore has obtained more popularity [13]. Combining two pharmacophore generation strategies has already become the mainstream in computer-aided virtual screening. Moreover, computational approaches such as molecular dynamics simulations, molecular docking, drugs-likeness prediction, and in silico ADMET studies are adopted mainly to screen potential drugs/molecules from various databases/libraries, saving experimental cost and time in drug discovery [14,15].

Starting from the Xanthohumol molecule and its proven anticancer activity from the literature [16,17,18,19,20], we performed virtual screening, ligand-based screening (ligand similarity), and structurally based (ligand docking on XIAP protein) screening. After building our pharmacophore model, molecular dynamics studies were used to evaluate the docking protocol. The best candidate molecules were also assessed through ADMET predictions to confirm their pharmacological use and quantum chemistry to evaluate their reactivity.

## 2. Computational Methods

After concluding from the literature the proven anticancer activity of Xanthohumol, we followed two approaches regarding the virtual screening procedure. First, using the SMILES chemical format to describe the structure of the starting molecule, we performed ligand-based screening similarity using the SwissSimilarity Webserver (http://www.swisssimilarity.ch/25/03/2022, accessed on 5 July 2022). Through the available screening libraries, we chose “ZINC drug” (a library of 10,639,400 structures) and, for the screening method, the “pharmacophore” build. This method yielded 400 candidate molecules. On the same server (SwissADME), pharmacokinetic property evaluation, physicochemical property evaluation, and drug-likeness were performed [21]. The pharmacokinetic scores were predicted using the online web application pkCSM (http://biosig.unimelb.edu.au/pkcsm/prediction, accessed on 5 July 2022). This procedure minimized the number of candidate molecules. Sixty-two candidate molecules had the characteristics to be considered as drugs based on our first structure (Xanthohumol). To further reduce the number of candidate molecules, structure-based screening was performed using AutoDock Vina (https://autodock.scripps.edu, accessed on 5 July 2022). Docking was carried out on PyRx using the AutoDock Vina option and ran at an ‘exhaustiveness’ of 8. The grid box was centered at X = 12.1477, Y = −3.5864, and Z = 18.4151, with a grid dimension of 45.0279 Å × 68.7439 Å × 56.9456 Å, thereby enclosing both the active site residues and the binding site. Following a series of ligand–receptor docking runs, Vina evaluated the results, calculated the binding affinities of the ligands, and clustered the resulting poses based on their conformational overlaps. After choosing the best pose from each group, the ligands were ranked according to their binding affinities [22]. According to their binding affinities, the docking results of the top ligands were first validated by re-docking them into the same defined regions of the receptor using AutoDock Vina. The re-docked complex was superimposed on the reference co-crystallized complex, and the root means square deviation (RMSD) was calculated.

Additionally, molecular dynamics simulations were performed as a second validation method, using the AMBER force fields [23]. The complexes were placed in a rectangular parallelepiped water box, and an explicit solvent model for water was used while the complexes were solvated with a 10 Å water cap. Chlorine ions were added as counterions to neutralize the system. Before the MD simulations, one step of minimization was carried out. Particle mesh Ewald electrostatics and periodic boundary conditions were used in the simulation [24]. The MD trajectories were run using the minimized structures as the starting conformations. The time step of the simulations was 2.0 fs with a cutoff of 10 Å for the non-bonded interactions. Constant-volume periodic boundary MD was carried out for 300 ps. The temperature was raised from 0 to 300 K. Then, 1.7 ns of constant-pressure irregular boundary MD was carried out at 300 K using the Langevin thermostat to maintain the temperature of our system constant. The ligand’s disposition was monitored. All ligands that showed an average RMSD of greater than 2 Å concerning the reference disposition were discarded using the docking result as a reference pose. 

The structure-based pharmacophore modeling was performed by molecular docking using the iGEMDOCK software [25]. The 5OQW coded crystal structure of XIAP protein was selected from the Protein Data Bank (www.rcsb.org, accessed on 5 July 2022). Ligand molecules (the best candidates with higher binding affinities) were collected by Drug Bank (www.drugbank.ca, accessed on 5 July 2022). Ligand molecules included the Xanthohumol molecule used for the pharmacophore modeling. The protein structure was then prepared by assigning the hydrogen atoms, charges, and energy minimization using CHIMERA software [26]. The energy minimization was performed using 500 steepest descent steps with a 0.02 Å step size and an update interval of 10. Before completing the molecular docking of ligand and receptor, the ligands were optimized by adding hydrogen using CHIMERA software. Using ORCA, DFT studies obtained the optimized structures under the B3LYP/6 311++G (d, p) level of theory [27,28,29]. The scoring function consisted of a simple empirical scoring function and a pharmacophore-based scoring function to reduce the number of false positives. The energy function can be dissected into the following terms:

E_tot_ = E_bind_ + E_pharma_ + E_ligpre_ (1)

E_bind_ is the empirical binding energy used during molecular docking; E_pharma_ is the energy of binding-site pharmacophores; E_ligpre_ is a penalty value if the ligand is unsatisfied with the ligand preferences. E_pharma_ and E_ligpre_ were used to improve the number of true positives. The empirical binding energy (E_bind_) is given as

E_bind_ = E_inter_ + E_intra_ + E_penal_ (2)

E_inter_ and E_intra_ are intermolecular and intramolecular energy, respectively. E_penal_ is a large penalty value if the ligand is out of the range of the search box. In this paper, E_penal_ was set to 10000. For screening: the population size was 200, the number of generations was 70, and the number of solutions was 3. Fitness is the total energy of a predicted pose in the binding site. The empirical scoring function of iGEMDOCK is estimated as

Fitness = vdW + H_bond_ + Elec. (3)

Here, the vdW term is van der Waals energy. H_bond_ and Elec terms are hydrogen bonding energy and electrostatic energy, respectively. Screenshots of the ligand–amino acid residue interactions were created by CHIMERA software. The docking results of the ligands were validated by re-docking them into the same defined regions of the receptor using the crystalized structure.

## 3. Results

**Figure 1:**
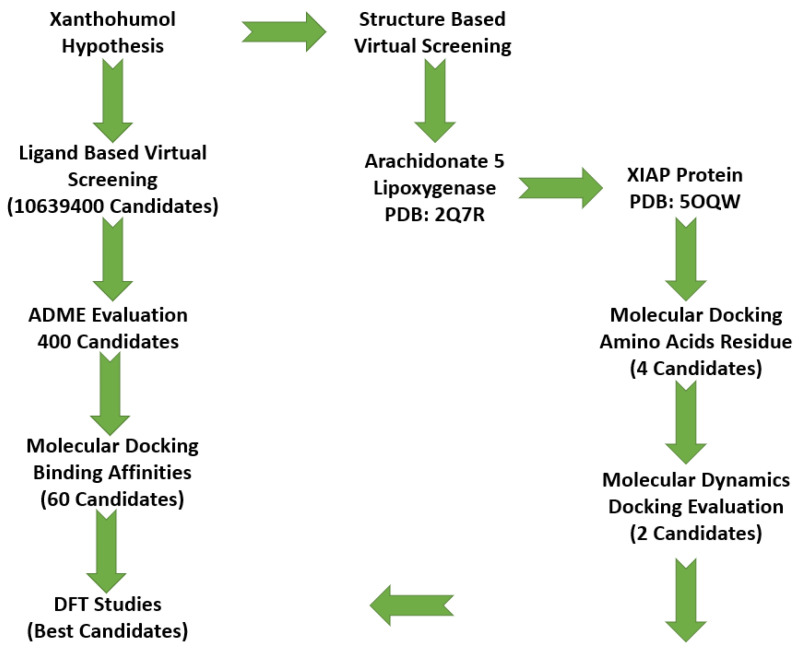
Workflow of the study.

**Figure 2:**
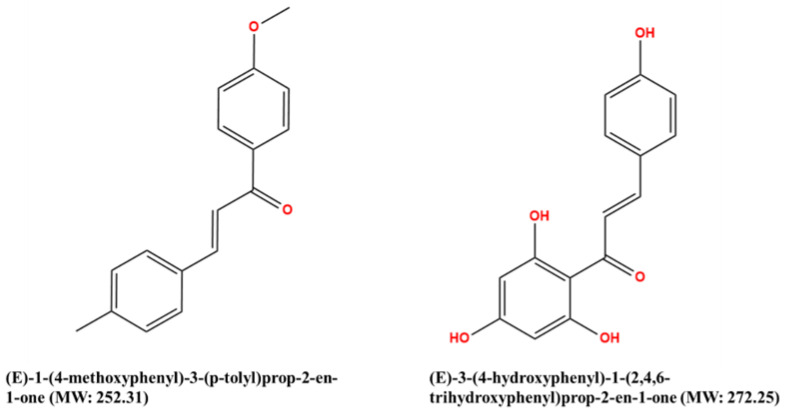
Candidate molecules.

**Figure 3:**
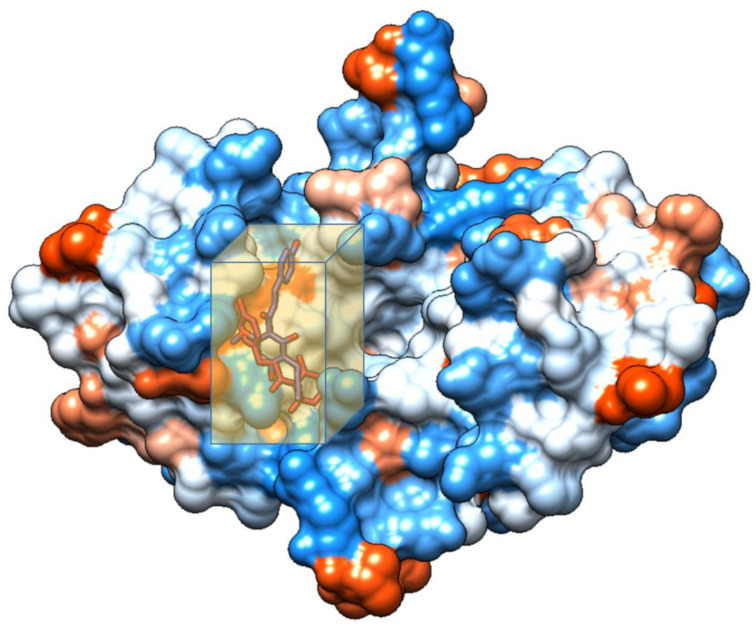
Binding pocket of XIAP protein.

**Figure 4:**
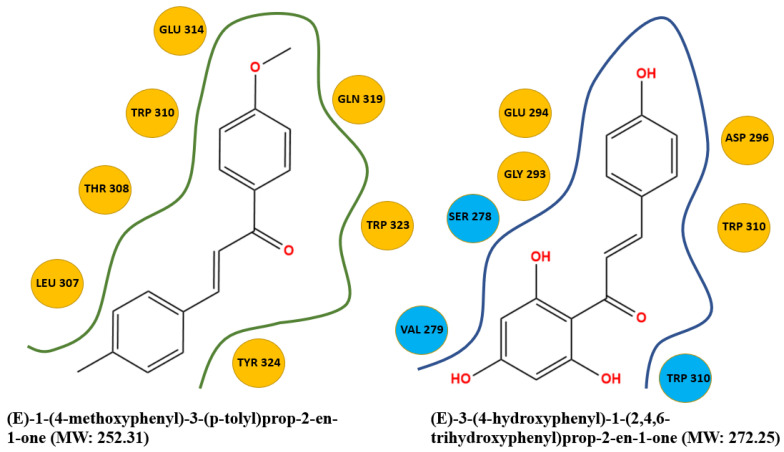
Amino acid residue of XIAP protein interacting with the two molecules (blue color: H-bonds).

**Figure 5:**
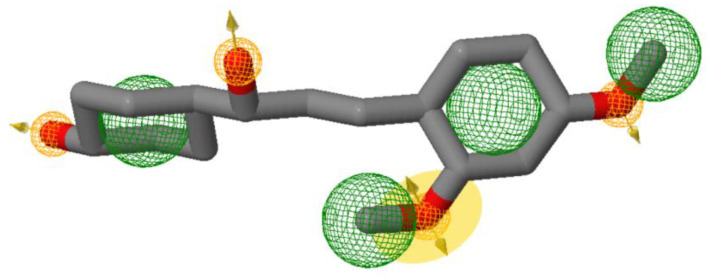
Pharmacophore descriptors.

**Figure 6:**
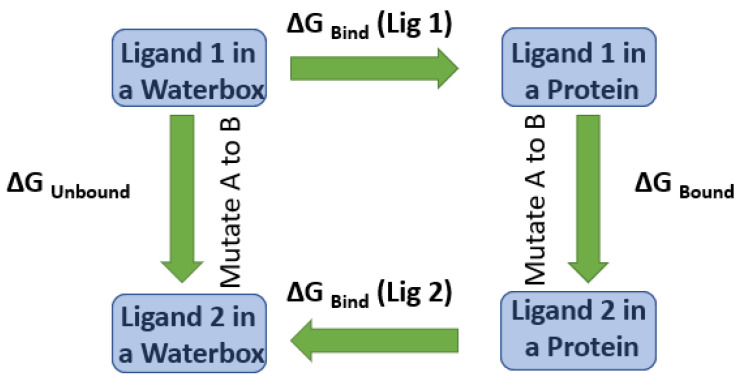
FEP calculates the difference in ΔG binding between Ligand 1 and Ligand 2 to a protein using alchemical changes to mutate A to B.

**Figure 7:**
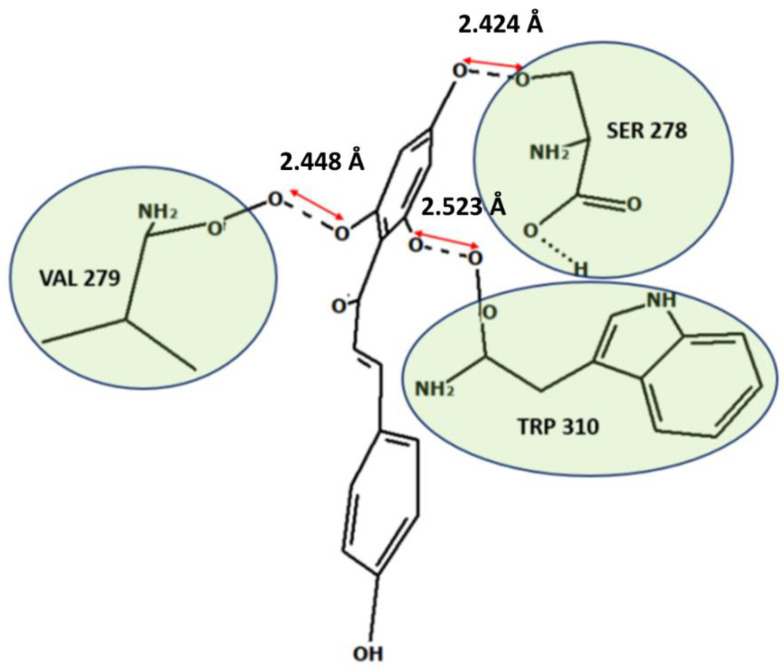
Hydrogen bonds description.

**Figure 8:**
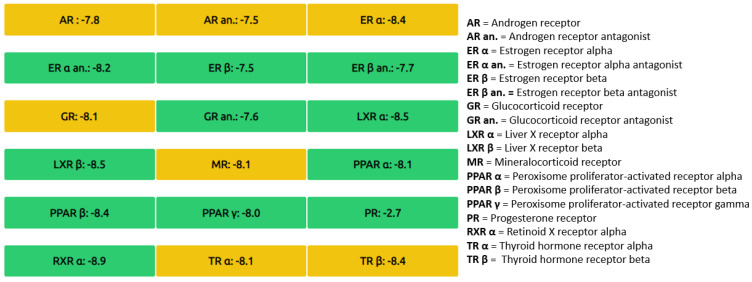
Endocrine disruption potential of the best candidate, compound 2, obtained from Endocrine Disruptome. Orange-yellow describes the medium probability of binding while the green color describes the low probability of binding.

After a literature search regarding the in vitro and in vivo anticancer activity of Xanthohumol, we realized that there was not any in silico procedure that evaluates or further studies this specific hypothesis. This prenylated chalcone provides a scaffold for other chalcone derivatives with the same or better anticancer activity. To verify this, using the Swiss Institute of Bioinformatics server, we found, based on this virtual ligand screening, that 10,639,400 similar structures could provide the same activity (SwissSimilarity). To minimize the number of candidate molecules, ADME studies, with the help of the same server, resulted in 400 that could be used as drugs. Additionally, these molecules can interact based on their chemical structure with the protein 5-lipoxygenase (SwissTargetPrediction) (2Q7R code for crystal structure in Protein Data Bank). Surprisingly, XIAP protein was not a candidate structure to interact with any of these molecules. XIAP is an antiapoptotic protein, and we believe that the inhibition of this protein could induce anticancer activity. Additional toxicity predictions were performed based on reference [30]. Furthermore, structure-based virtual screening was followed for these two protein structures (2Q7R and 5OQW). The ligands were the 400 candidate molecules derived after ADME studies. Sixty-two of these molecules, based on their binding affinities (with 5-lipoxygenase) after docking studies, were used in further docking studies with the XIAP protein (5OQW). The docking results were evaluated with the methods described above in computational methods. The complete flowchart of the work can be seen in **Figure 1**. The two best candidate molecules, (E)-1-(4-methoxyphenyl)-3-(p-tolyl) prop-2-en-1-one (MW: 252.31) **(A)** and (E)-3-(4-hydroxyphenyl)-1-(2,4,6-trihydroxyphenyl) prop-2-en-1-one (MW: 272.25) **(B)**, are depicted in **Figure 2**. After evaluating the docking protocol, the two molecules were selected (RMSD value < 2 Å). Docking validation RMSD values can be found in Supplementary Figures S1 and S2 for the two molecule candidates. The RMSF study value for E)-3-(4-hydroxyphenyl)-1-(2,4,6-trihydroxyphenyl) prop-2-en-1-one can also be found in **Figure S3**. The RMSF defines the deviation of the particle in the protein (XIAP). The residues with the higher peaks belong to the loop areas of the protein. On the other hand, the stability of the ligand binding to the protein is shown by the low RMSF values of binding site residues [30]. Density functional theory studies on B3LYP/6 311++G (d, p) were performed to discriminate the chemical reactivity between (E)-1-(4-methoxyphenyl)-3-(p-tolyl) prop-2-en-1-one and (E)-3-(4-hydroxyphenyl)-1-(2,4,6-trihydroxyphenyl) prop-2-en-1-one. We were able to calculate the molecular orbitals of the two molecules as well. The value of the energy difference between HOMO and LUMO and the highest occupied molecular orbital (EHOMO) and lowest unoccupied molecular orbital (ELUMO) energies play a significant role in the stability and reactivity of molecules. The EHOMO energies of molecules show the molecule’s ability to donate electrons. On the other hand, ELUMO characterizes the ability of the compound to accept electrons. Electronegativity (*χ*) measures an atom’s power to attract a bonding pair of electrons. Based on equation *χ* = −(EHOMO + ELUMO)/2, a larger Δgap always indicates lower chemical reactivity and higher kinetic stability of the investigated species. The simultaneous effect of different parameters causes the chemical reactivity of molecules. The distribution and energy of HOMO are important parameters to explain the antioxidant potential of phenolic antioxidants. The electron-donating capacity of the molecule can be predicted by looking at the energy values of HOMO. The value of the energy difference between HOMO and LUMO, as well as the highest occupied molecular orbital (EHOMO) and lowest unoccupied molecular orbital (ELUMO) energies, plays a critical role in stability and reactivity [31]. In particular, in the first candidate, the LUMO orbital equals −6.243 eV while the HOMO orbital equals −11.202 eV. On the other hand, regarding the second molecule, the LUMO orbital equals −5.487 eV, and the HOMO orbital equals −10.855 eV. Based on that, the **B** molecule is more electronegative than the **A** (larger Δgap). The quantum chemical descriptors of the molecules can be found in **Table 1**. The results show that (E)-3-(4-hydroxyphenyl)-1-(2,4,6-trihydroxyphenyl) prop-2-en-1-one has higher reactivity based on the calculated energy gap of the HOMO and LUMO orbitals. These results are in agreement with the docking work. The docking results show that (E)-3-(4-hydroxyphenyl)-1-(2,4,6-trihydroxyphenyl) prop-2-en-1-one has better binding affinity amongst the best two candidates (−72.13 Kj/mol). Specifically, molecule **A** has a binding affinity of −69.10 kJ/mol with the target protein. Additionally, this energy corresponds only to van der Waals interactions since the molecule has no hydrogen bond with the amino acid pocket. The amino acid residue of the protein that interacts with molecule **A** is Leu 307, Thr 308, Trp 310, Glu 314, Gln 319, Trp 323, and Tyr 324. Molecule **B** interacts with three hydrogen bonds with Ser 278, Val 279, Trp 310 (energy contribution, −12.08 Kj/mol), and has van der Waals interactions with Val 279, Gly 293, Glu 294, Asp 296, and Trp 310 (energy contribution −62.05 Kj/mol). The total binding affinity of the **B** molecule with the XIAP protein is −74.13 Kj/mol. Docking results can be found in **Table 2**. Additionally, the binding position of the best conformations on the XIAP protein can be seen in **Figure 3**. Here, we can see that the conformations of the best candidates interact with the binding pocket of the XIAP protein. In **Figure 4**, we can see the detailed interaction of the amino acid residues of the XIAP protein with (E)-1-(4-methoxyphenyl)-3-(p-tolyl) prop-2-en-1-one and (E)-3-(4-hydroxyphenyl)-1-(2,4,6-trihydroxyphenyl) prop-2-en-1-one. ADME studies were repeated in particular for (E)-3-(4-hydroxyphenyl)-1-(2,4,6-trihydroxyphenyl) prop-2-en-1-one since it has the best binding affinity with the protein; the results are depicted in **Table 3**. The best candidate (Molecule **B**), in total, has 20 heavy atoms (no hydrogen atoms) and three rotatable bonds. The important aspect is that it has five hydrogen bond acceptors and four hydrogen bond donors. The interaction with the XIAP protein created three hydrogen bonds with the amino acids Ser 278, Val 279, and Trp 310. It passes all Lipinski’s rules to be regarded as a drug and does not penetrate the blood–brain barrier, an important aspect in its future use as an anticancer agent. In **Table 4** we can see additional toxicological information. Our candidate does not seem to be hepatotoxic nor cardiotoxic (70% confidence). In addition, it does not cause skin sensitization and the maximum tolerated dose is 0.373 (log mg/Kg/day). Drug–medication interactions may occur when three cytochrome isoforms are inhibited. In our case, CYP2C19 is not inhibited, while the other two isoforms are. Regarding the P-gp, our candidate chalcones are not sensitive to the efflux mechanism of P-gp. This is an indicator that our candidate molecules will probably not develop resistance to cancer cell lines. A bioavailability score of 0.55 additionally confirms good absorption after oral administration. Finally, the pharmacophore descriptors, hydrogen donor atoms, and hydrogen acceptor atoms can be found in **Figure 5**, while details about the radius and coordinates are Supplementary in **Table S1**. Specifically, we can observe the positions of the molecules that are responsible for hydrogen bonding with the XIAP protein and the areas responsible for the van der Waals interactions. After using ligand- and structure-based virtual screening, we present two possible candidates based on the prenylated chalcone Xanthohumol and the X-linked IAP antiapoptotic protein, the ((E)-1-(4-methoxyphenyl)-3-(p-tolyl) prop-2-en-1-one and the (E)-3-(4-hydroxyphenyl)-1-(2,4,6-trihydroxyphenyl) prop-2-en-1-one), which can be considered in further anticancer in vitro and in vivo studies. The use of quantum chemistry through density functional theory studies showed evidence of the higher reactivity of (E)-3-(4-hydroxyphenyl)-1-(2,4,6-trihydroxyphenyl) prop-2-en-1-one), given the fact that it is in agreement with the better free Gibb’s energy of the stable complex with the XIAP protein. Free energy perturbation calculations for the two chalcone derivatives for the hydrogen bond probability to occur were performed. In **Figure 6** we represent a comprehensive theory behind the calculation, indicating how the binding energies were corrected after the ligands were rearranged with solvent (water) molecules. A detailed explanation of the calculating procedure can be found in references [31,32,33,34,35]. The error calculated in the binding affinities of the best two candidates was less than 4 KJ/mol, indicating that molecule 2 still remains strongly bound to the XIAP protein and is a good inhibitor candidate. Additional density functional theory studies were performed to evaluate the hydrogen bond creation of the best molecule candidate with the amino acids valine 279, serine 278, and tryptophane 310 of the XIAP protein. The studies revealed the distance of the hydrogen bond with valine 2.448 Å, with serine 2. 424 Å, and with the tryptophane 2. 523 Å (**Figure 7**). The multi colored table shown in **Figure 8** was obtained from the online web tool of Endocrine Disruptom (http://endocrinedisruptome.ki.si, accessed on 5 July 2022). These fourteen nuclear receptors, with eighteen targets, show the binding probability of our candidate molecule. The green color indicates a low probability (sensitivity > 0.75), the yellow-orange color indicates a medium probability (0.50 < sensitivity < 0.75) of binding, and the red color (which is absent here) indicates a high probability (sensitivity < 0.25). The low to medium probability binding in these receptors indicates a strong profile of our candidate chalcone molecule. 

## 4. Discussion

This work provided some interesting data regarding the anticancer possibilities of chalcone molecules. The hypothesis started with Xanthohumol. Xanthohumol is a prenylated chalcone derived from hops and can be found in beer. In recent years, its anticancer and antioxidant activities have become well known and have also been studied in vitro and in vivo. At the same time, the role of the XIAP protein has been established in the bibliography, but surprisingly, there were not any studies connecting the anticancer potential of Xanthohumol with the XIAP protein. The same interesting fact was revealed in structure similarity studies online, using the best candidates of the ligand-based virtual screening studies. There was not any connection between the chalcone moieties and the inhibition of the activity of the XIAP protein. Our studies suggest that the inhibition of the XIAP protein could promote anticancer activity and, at the same time, our lead candidates, (E)-1-(4-methoxyphenyl)-3-(p-tolyl) prop-2-en-1-one and (E)-3-(4-hydroxyphenyl)-1-(2,4,6-trihydroxyphenyl) prop-2-en-1-one, would form strong complexes with the target protein and could be possible inhibitors of its antiapoptotic activity. Furthermore, we could not propose these candidates before evaluating them in silico according to their ADMET properties. Interestingly, these molecules passed the Lipinski’s rule of five and, at the same time, were neither cardiotoxic (70% confidence) nor nephrotoxic, and can be used further as drug candidates. Our final approach was to compare these two compounds in terms of density functional theory studies and concluded that (E)-3-(4-hydroxyphenyl)-1-(2,4,6-trihydroxyphenyl) prop-2-en-1-one (the second molecule) has more advantages compared to the (E)-1-(4-methoxyphenyl)-3-(p-tolyl) prop-2-en-1-one (the first one). These advantages have to do with their reactivity and chemical stability, a fact that was in a good agreement with docking studies, since the second molecule formed stronger complexes with the target protein. In this study, we used all of the known in silico techniques to obtain our results: molecular docking, molecular dynamics, density functional theory studies, ADMET studies, and FEP correction of the binding constants. We promoted the hypothesis of the XIAP antiapoptotic protein as a good target for anticancer activity and, at the same time, we promoted two chalcone compounds that could be used more in vivo and in vitro for cancer therapy. The candidates were chosen from 10639400 compounds, and only a few weeks were needed to gather this information. Several more weeks were needed to evaluate and distribute the information in a scientific matter; however, this was nothing compared to years of laboratory work to gather such findings. That is not to mention the resources and costs that have been saved using this in silico procedure. Technological improvements in the field of computer-aided drug design and discovery need to be used each time a drug discovery project commences, before the actual experimental procedures. This will save cost and time; therefore, important drugs could enter the market much sooner, saving hundreds of patients fighting diseases. 

## 5. Conclusions

Based on computer-aided drug discovery (CADD) calculations, we predicted two chalcone molecules as good candidates to be evaluated as inhibitors for the antiapoptotic protein XIAP. The calculations provide new insights into anticancer drug discovery since XIAP is highly expressed in cancers of diverse tumor types and is considered an attractive therapeutic target. We built our pharmacophore model based on the Xanthohumol hypothesis. XN is a biomolecule with proven in vitro and in vivo anticancer activity, and we ran ligand-based and structure-based virtual screening. Starting from 10639400 structures, we concluded that ((E)-1-(4-methoxyphenyl)-3-(p-tolyl) prop-2-en-1-one and (E)-3-(4-hydroxyphenyl)-1-(2,4,6-trihydroxyphenyl) prop-2-en-1-one) are the best candidates. Moreover, quantum chemical descriptors help us to understand and discriminate the second molecule as a better structure due to its higher chemical activity. (E)-3-(4-hydroxyphenyl)-1-(2,4,6-trihydroxyphenyl) prop-2-en-1-one) passed Lipinski’s rule for drugs; it has five hydrogen acceptor atoms and four hydrogen donor atoms, making it easy to create hydrogen bonds with the amino acids Ser 278, Val 279, and Trp 310 of the XIAP binding pocket. 

## Figures and Tables

**Table 1:** Quantum chemical descriptor of the best candidates calculated by density functional theory study.

Quantum Chemical Descriptor	(E)-1-(4-Methoxyphenyl)-3-(p-tolyl) prop-2-en-1-one	(E)-3-(4-Hydroxyphenyl)-1-(2,4,6-trihydroxyphenyl) prop-2-en-1-one
*μ*	−8.723 eV	−8.171 eV
*n*	4.959 eV	5.386 eV
*I*	11.202 eV	10.855 eV
*A*	6.243 eV	5.487 eV
*ω*	7.672 eV	6.198 eV
*χ*	8.732 eV	8.171 eV
*ζ*	0.202 eV	0.186 eV
*E_gap_*	4.959 eV	5.386 eV

**Table 2:** Energies and amino acid residue of the best two candidates on the XIAP protein.

Complex	Total Energy (KJ/mole)	Energy H_Bond_ (KJ/mole)	Energy VDW (KJ/mole)	Amino Acid Residue H_Bond_	Amino Acid Residue VDW Interactions
A-5OQW	−69.10	0	−69.10	None	Leu 307, Thr 308, Trp 310, Glu 314, Gln 319, Trp 323, Tyr 324
B-5OQW	−74.13	−12.08	−62.05	Ser 278, Val 279, Trp 310	Val 279, Gly 293, Glu 294, Asp 296, Trp 310

**Table 3:** ADME studies of the second molecule (best candidate).

ADME Characteristics	Value-Answer
Formula	C_15_H_12_O_5_
Molecular weight	272.25 g/mol
Number of heavy atoms	20
Number of rotatable bonds	3
Number of Hydrogen bond acceptors	5
Number of Hydrogen bond donors	4
Molar refractivity	74.34
Log P_o/w_	1.90
Log S	−3.55
GI absorption	High
BBB permeant	No
CYP1A2 Inhibitor	Yes
CYP2C19 Inhibitor	No
CYP2C9 Inhibitor	Yes
CYP2D6 Inhibitor	No
CYP3A4 Inhibitor	Yes
Log Kp	−5.96 cm/s
Lipinski	Yes, 0 violation
Bioavailability Score	0.55
Lead-likeness	Yes
Synthetic accessibility	2.56
Pg Substrate	No

**Table 4:** Toxicity results of the second molecule (best candidate).

Description	Prediction
AMES Toxicity	No
Maximum Tolerated Dose	0.373 (log mg/Kg/day)
hERG I Inhibitor	No
hERG II Inhibitor	Yes
Oral Rat Acute Toxicity (LD50)	2.193 (mol/Kg)
Oral Rat Chronic Toxicity (LOAEL)	2.690 (log mg/Kg_bw/day)
Hepatotoxicity	No
Skin Sensitization	No
*T. Pyriformis* Toxicity	0.318 (log ug/L)
Minnow Toxicity	0.752 (log mM)
Non Cardiotoxic	70% Confidence

## Data Availability

Not applicable.

## Supplementary Materials

The following supporting information can be downloaded at: https://www.mdpi.com/article/10.3390/molecules27154825/s1, Figure S1: RMSD value for (E)-1-(4-methoxyphenyl)-3-(p-tolyl) prop-2-en-1-one during simulation time. Figure S2: RMSD value for (E)-3-(4-hydroxyphenyl)-1-(2,4,6-trihydroxyphenyl) prop-2-en-1-one during simulation time. Figure S3: RMSF value for (E)-3-(4-hydroxyphenyl)-1-(2,4,6-trihydroxyphenyl) prop-2-en-1-one during simulation time. Table S1: Pharmacophore model.
