# Supplementary material for: Ligand and Structure-Based Virtual Screening in Combination, to Evaluate Small Organic Molecules as Inhibitors for the XIAP Anti-Apoptotic Protein: The Xanthohumol Hypothesis
Source: Molecules. 2022 Jul 28;27(15):4825. doi: 10.3390/molecules27154825 (PMC9369490; doi:10.3390/molecules27154825)
Supplement: Supplementary file 1 [file molecules-27-04825-s001.zip › molecules-1798949-supplementary.pdf]

Supplementary Material

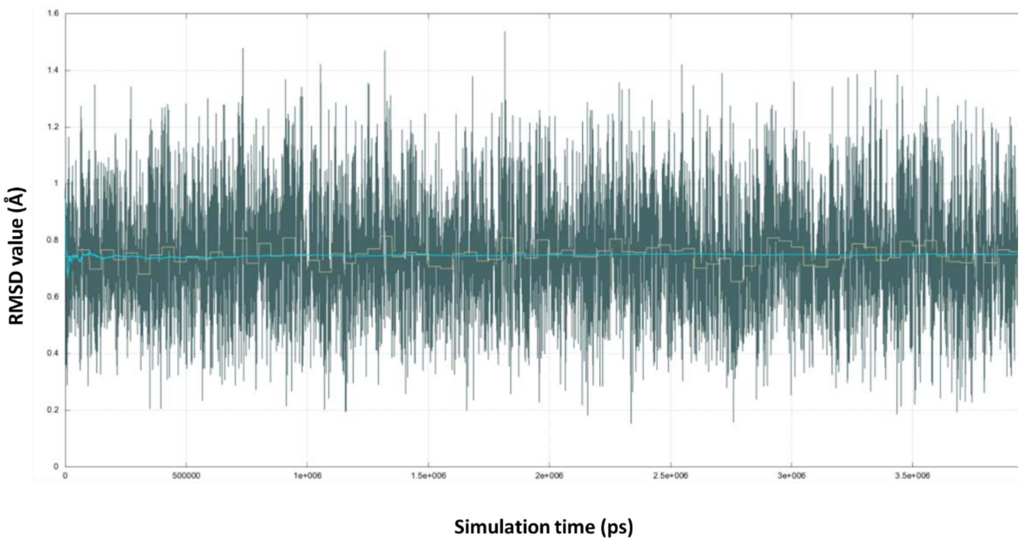

Figure S1: RMSD value for (E)-1-(4-methoxyphenyl)-3-(p-tolyl) prop-2-en-1-one during simulation time.

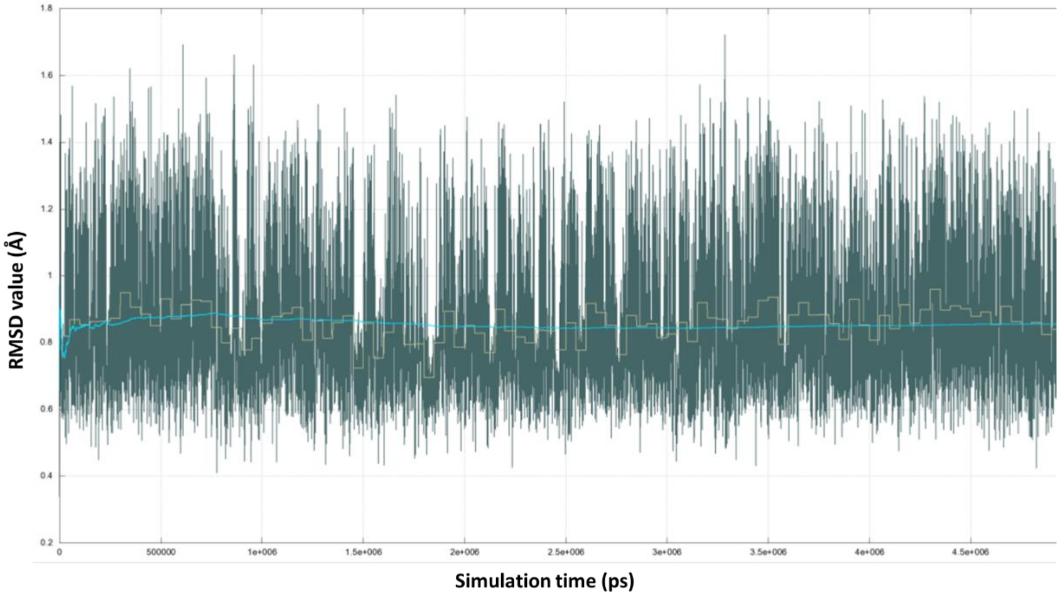

Figure S2: RMSD value for (E)-3-(4-hydroxyphenyl)-1-(2,4,6-trihydroxyphenyl) prop-2-en-1-one during simulation time.

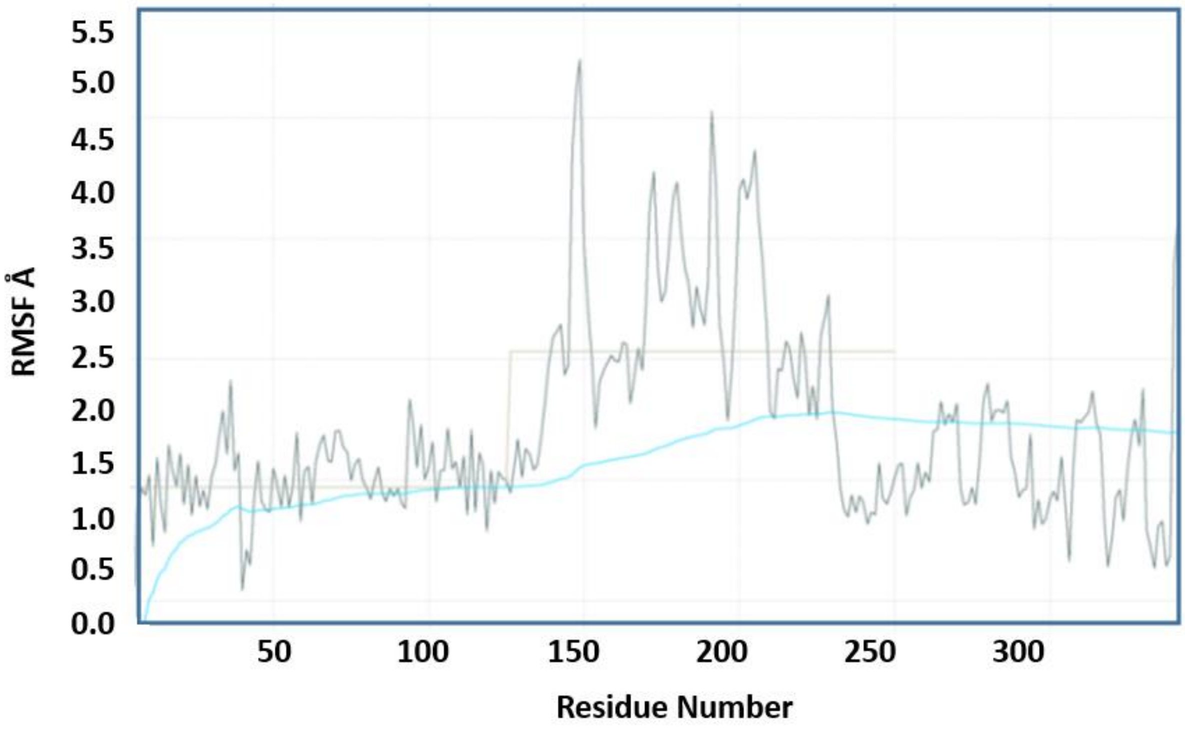

Figure S3: RMSF value for (E)-3-(4-hydroxyphenyl)-1-(2,4,6-trihydroxyphenyl) prop-2-en-1-one during simulation time.

Table S1: Pharmacophore model.

x	y	z	Radius	Enabled
Hydrogen Acceptor	50.3	63.89	7.03	0.5
Hydrogen Acceptor	53.19	67.16	5.08	0.5
Hydrogen Acceptor	53.1	59.29	7.83	0.5
Hydrogen Acceptor	51.05	58.12	13.78	0.5
Hydrogen Acceptor	53.1	59.29	7.83	0.5
Hydrogen Donor	51.05	58.12	13.78	1
Hydrogen Donor	50.1	62.51	7.11	1
Hydrogen Donor	54.32	67.29	4.26	1
Hydrogen Donor	52.78	64.59	6.02	1
Hydrophobic	51.6	59.05	11.23	1
